# Differences in incubation behaviour and niche separation of two competing flycatcher species

**DOI:** 10.1007/s00265-020-02883-4

**Published:** 2020-08-01

**Authors:** Tuuli-Marjaana Koski, Päivi M. Sirkiä, S. Eryn McFarlane, Murielle Ålund, Anna Qvarnström

**Affiliations:** 1 Department of Biology and Biodiversity Unit, University of Turku, FI-20014 Turku, Finland; 2 Integrated Plant Protection Unit, Department of Plant Protection Biology, Swedish University of Agricultural Sciences, SE-230 53, Alnarp, Sweden; 3 Finnish Museum of Natural History, Zoology Unit, University of Helsinki, P.O. Box 17, FI-00014 Helsinki, Finland; 4 Institute of Evolutionary Biology, University of Edinburgh, Charlotte Auerbach Road, Edinburgh, EH9 3FL UK; 5 Biological Sciences, Lund University, Sölvegatan 37, SE-223 62 Lund, Sweden; 6 Department of Integrative Biology, Michigan State University, 288 Farm Lane, East-, Lansing, 48824 USA; 7 Animal Ecology, Department of Ecology and Genetics, Uppsala University, Norbyvägen 18d, SE-752 36 Uppsala, Sweden

**Keywords:** Incubation behaviour, Flycatcher, Niche separation, Food availability, Interspecific competition

## Abstract

**Abstract:**

Food availability sets the stage for incubation behaviour of a female bird and thereby indirectly determines the nest temperature, which in turn affects development and metabolism of avian embryos. Changes in development and metabolism in turn are known to influence offspring’s ability to adjust to environmental changes later in life. However, few studies have investigated the role of interspecific differences in incubation behaviour in relation to niche separation between competing sibling species. We studied the effects of habitat quality (in terms of caterpillar availability) on incubation behaviour of two ecologically similar and closely related species, collared and pied flycatchers (*Ficedula albicollis* and *F. hypoleuca*), in their hybrid zone on the island of Öland, Sweden. Even though both species prefer caterpillar-rich deciduous forests as nesting sites, collared flycatchers, whose nestlings have higher energetic demands, are able to nest only in deciduous forests, whereas pied flycatchers have more flexible habitat requirements. Overall, higher food availability was associated with increased nest attendance, higher incubation temperature and a lower number of foraging trips across species. In addition, collared flycatchers had more frequent and shorter foraging trips across habitat types, allocated more heat to eggs and therefore maintained higher nest temperatures compared to pied flycatchers. We argue that the higher heat allocation or the need to maintain a higher nest temperature for embryo development may constrain collared flycatchers to focus on relatively more profitable prey. Our results highlight the importance of considering incubation behaviour in the context of understanding species differences in niche use.

**Significance statement:**

Niche separation plays an important role in mitigating effects of competition between closely related species. Whether species differences in incubation behaviour relate to differences in niche use remains unknown. We compared incubation behaviour of two sympatric flycatcher species that differ in sensitivity to food availability. The competitively more dominant and larger species, the collared flycatcher, whose nestlings are more sensitive to food shortages, made more frequent foraging trips but allocated more heat to eggs, leading to higher nest temperature despite lower nest attendance, compared to pied flycatchers. These interspecific differences may be a result of differences in embryo sensitivity or female physiology and contribute to the niche separation between the species, which in turn can facilitate coexistence.

**Electronic supplementary material:**

The online version of this article (10.1007/s00265-020-02883-4) contains supplementary material, which is available to authorized users.

## Introduction

Incubation is an important part of parental investment for birds, because by altering the egg thermal environment, the parent not only influences the hatching success and timing but also the later viability and competitive ability of the offspring by affecting, for example, their metabolism and phenotype (Hepp et al. 2006; Olson et al. 2006; Ardia and Clotfelter 2007; Wada et al. 2015; Nord and Nilsson 2011, 2016; Mueller et al. 2019). Incubation is energetically costly, because the parent (especially in the case of uniparental incubation) must trade off the maintenance of a suitable thermal environment for embryo development against the fulfilment of its own energetic needs (Haftorn 1988; Conway and Martin 2000; Reid et al. 2000a; Reid et al. 2002; Hainsworth and Voss 2002; Cooper and Voss 2013). In fact, the energetic demand of an incubating parent can be as high as during nestling provisioning (Thomson et al. 1998), and may even reduce the later provisioning performance (Heaney and Monaghan 1996). Therefore, it is not surprising that balancing between self-maintenance and incubation is especially challenging when food availability is low. For example, poor quality female tree swallows (*Tachycineta bicolor*) and pied flycatchers (*Ficedula hypoleuca*) allocate more time to self-maintenance and thus less time to incubating, resulting in lower nestling quality or longer incubation periods compared to higher quality females (Lifjeld and Slagsvold 1986; Ardia and Clotfelter 2007). Conversely, in high-quality territories, birds are more likely able to take fewer or shorter *off*-*bouts*, i.e. foraging trips out of the nests, and have longer *on*-*bouts*, i.e. nest-attendance periods between off-bouts (Drent et al. 1985; Eikenaar et al. 2003; Rastogi et al. 2006; Ardia and Clotfelter 2007; Amininasab et al. 2016; Vafidis et al. 2018). This in turn should affect hatching success, offspring quality and/or the likelihood for the offspring surviving to adulthood due to more constant or higher incubation temperatures, and/ or shorter incubation periods (number of days incubated before hatching), (Ardia and Clotfelter 2007; Nord and Nilsson 2011, 2016; Mueller et al. 2019).

Although closely related species often share similar ecological requirements, they can differ in their ability to respond to environmental stressors, such as changes in food availability, due to subtle differences in physiology and behaviour. For example, the collared flycatcher (*Ficedula albicollis*) and the closely related species, pied flycatcher, share a preference for deciduous forests as breeding sites in their hybrid zone on the Swedish island, Öland. However, pied flycatcher nestlings are better able to withstand limited food compared to the larger and dominant collared flycatcher. Pied flycatchers are thus able to breed in a wider variety of habitats, including the less favoured pine forests, whereas the higher sensitivity to food availability limits the breeding of collared flycatchers to high-quality deciduous habitats (Qvarnstörm et al. 2005; Qvarnström et al. 2007; Rybinski et al. 2016; Sirkiä et al. 2018). Further, young pied flycatchers are often displaced from the preferred deciduous forests to mixed- and coniferous forest territories by collared flycatchers (Lundberg and Alatalo 1992; Qvarnstörm et al. 2009; Veen et al. 2010; Vallin et al. 2012a, b). Pied flycatcher nestlings also are better able to adjust their metabolism according to environmental changes (McFarlane et al. 2018), which may partially explain their higher tolerance to low food availability. As incubation behaviour and thus the thermal environment during embryo development influences several physiological and developmental features of the off-spring, both in the short and longer term, it may thus also partly influence specie’s competitive ability and capacity to adapt to environmental changes. We tested this by investigating whether the two competing flycatcher species differ in incubation behaviour, and by studying the role of habitat-specific food availability on nest attendance, both of which may contribute to the differences in competitive abilities and niche use described above.

We focused on the caterpillars as a measure of habitat quality as caterpillars are the most important food source for collared and pied flycatchers (Arnold et al. 2010; Burger et al. 2012). We predicted that the females of both species would reduce the number, or shorten the length of foraging trips with increasing habitat quality, resulting in longer incubation sessions, higher incubation constancy and temperatures, and consequently, increased hatching success. Moreover, because of the lower tolerance of collared flycatchers to variation in habitat quality, we predicted that (1) incubating collared flycatcher females would be more sensitive to lower food availability requiring more frequent or longer foraging trips during incubation (especially in poorer quality habitats). This should result in lower nest temperatures in collared flycatcher nests compared to pied flycatchers, as we expected the latter species to require fewer or shorter foraging trips. Further, collared flycatcher embryos may also be less tolerant to lower incubation temperatures (resulting from lower nest attendance) compared to pied flycatcher embryos, and thus collared flycatcher females may need to compensate lower nest attendance by maintaining higher incubation temperatures across habitat types. Alternatively, if pied flycatcher females are not tolerant to lower food availability but instead allocate more resources to foraging, but their embryos are relatively more tolerant to lower nest attendance, we expect that the hatching success of pied flycatchers would not be influenced by the resulting lower nest temperature.

## Methods

### Species and field site

Flycatchers are small insectivorous birds that overwinter in Africa and arrive at the breeding grounds in northern Europe in late April and early May and start defending natural breeding holes or nest boxes (Lundberg and Alatalo 1992; Pärt and Qvarnström 1997). Females lay five to seven eggs that hatch in the beginning of June. Both parents feed their offspring with insects for approximately 2 weeks in the nest and 2 weeks after fledging (Lundberg and Alatalo 1992; Qvarnstörm et al. 2009). Caterpillars are a highly nutritious and important food source for flycatchers during this nestling provisioning period (Arnold et al. 2010; Eeva et al. 2010) and can contribute up to 80% of the nestling diet (Burger et al. 2012). Flycatcher males can feed the incubating females during harsh conditions but typically stop if the female is able to find enough prey on her own (Lifjeld and Slagsvold 1986; Cantarero et al. 2014; Amininasab et al. 2016; Kötél et al. 2016).

The relative proportion of breeding pied flycatchers varies from less than 10 to 100% across the different woodlots on Öland (Rybinski et al. 2016). The breeding area consists of a mixture of agricultural land and various-sized areas of deciduous forest, where we have established more than 20 separate nest box plots, each consisting of 25–350 boxes (Qvarnstörm et al. 2009; Rybinski et al. 2016). The most common tree species found in our study area are oak (*Quercus robur*), hazel (*Corylus avellana*), ash (*Fraxinus excelsior*) and birch (*Betula pendula*). Some coniferous forests can be found in the far northern part of the island and are dominated by pine (*Pinus sylvestris*) (Qvarnstörm et al. 2009). In the deciduous habitats, the caterpillar availability is high and occurs earlier in the spring with sharp decline in availability across the season. In contrast, caterpillar availability is lower but more stable in coniferous forests (Veen et al. 2010; Vallin et al. 2012a; Rybinski et al. 2016). Among breeding pairs sampled in this study, the average habitat quality was similar in territories occupied by collared and pied flycatchers (raw data presented in Supplement 1, Fig. S1).

### Population monitoring and habitat quality

Data on incubation behaviour (between 19 May and 6 June), breeding success as well as habitat quality was collected in 2014 from 44 nests (collared flycatchers *N* = 30, pied flycatchers *N* = 14). Out of these, 11.36% (i.e. five nests, all pied flycatchers) were located in pine forests, whereas the rest of the flycatcher nests were located in deciduous habitats. The breeding success of flycatchers was monitored until fledging, ending in early July. Both species breed in standard wooden nest boxes (29 × 10 × 10 cm). Incubation was expected to start after the female laid her final egg (incubation day 0). Nests were chosen for this study to display variation in habitat quality and laying date in both species. Temperature data loggers (wireless Ibutton 1-Wire/iButton, model DS1922L-F5 with temperature accuracy of ± 0.5 °C from − 10 to + 65 °C) were set to record the temperature once per minute, with a resolution of 0.0625 °C (Nord and Nilsson 2012). The loggers were placed under the eggs, in the centre of the nest cup during the early incubation stage (on average 1 day after laying the last egg, varying from 0 to 5 days), and replaced with a new logger during the middle stage of incubation (on average 6 days after laying the last egg, varying from 5 to 8 days).

As flycatchers arrange the eggs around the logger, enabling the logger to be in direct contact with the brood patch, the measurement method has been shown to adequately reflect female heat transfer to the clutch in pied flycatcher (Nord and Nilsson 2012). The loggers were placed into the nests around midday, after which they recorded the temperature for ca. 68 h. At day 6 of incubation, females were captured with swing door traps, identified to species, ringed, weighted and their tarsus measured (to the nearest dg and mm, respectively). After the last egg is laid, flycatchers incubate on average for 12 days (Lundberg and Alatalo 1992), and hatching date was thus predicted as lay date + (number of eggs + 12 days). The hatch checks were done around midday-afternoon of the expected hatching date, and repeated daily until the first egg hatched. The average clutch size was 6.6 (SD 0.67) and it did not differ between the two flycatcher species (ANOVA *F*_1, 42_ = 1.26, *P* = 0.27), but since clutch size is known to significantly affect incubation behaviour and/or nest temperature (e.g., Reid et al. 2000b; Nord and Nilsson 2012), it was included as a covariate in the statistical models (see the “Statistical analyses” section).

Habitat quality data was handled following the methods described in Rybinski et al. (2016). In short, we first measured caterpillar abundance associated with 12 most common tree species (*N* = 43 tree individuals) in the study area using custom-made collectors of frass (i.e. caterpillar faecal pellets) placed under the canopy. Because frass abundance is dependent on the canopy size, the heights of tree crowns were also measured and the dry mass of the frass was converted into an average of frass mg/day/m^3^ of canopy for each tree species. Secondly, we estimated the tree species composition around each nest box by using a relascope, which takes into account the distance of each tree from the observer, and the size of the trunk. Trunk size, in turn, is correlated with the canopy size and consequently, the caterpillar abundance. Based on the information about the tree species, canopy sizes around the nest boxes as well as frass measurements associated with each tree species, we calculated point estimates of habitat quality around the nest boxes. However, the feeding trips of the flycatchers are known to be larger than the areas covered by the point estimates and we therefore used a weighted average of all point estimates within a radius of 150 m for each nest. This way, we could account for caterpillar abundance for each tree species, the tree species composition around each nest and the canopy size of each tree (i.e. tree size) around the nest, to get a comprehensive estimate of caterpillar availability in a given territory. This gave an estimate of the food availability in the foraging range of each breeding pair of flycatchers (Rybinski et al. 2016). As the caterpillar availability has temporal variation within the habitat type (Veen et al. 2010; Vallin et al. 2012a; Rybinski et al. 2016), we calculated habitat quality values separately for the early and middle incubation stage for each nest as a weighted average.

### Incubation data

The off-bouts (seen as a continuous drop in temperature) were identified from temperature logger data using the program Rhythm, which allows for visual detection of off-bouts in the program Raven (Cooper and Mills 2005). Following the previously used parameters for investigating incubation behaviour of pied flycatchers (Nord and Nilsson 2012), the minimum off-bout length was set to 4 min and minimum temperature decrease of 1 °C. In addition, cooling rate was set to 0.15 °C/min. The selections were checked and corrected if needed by an observer who was blind to the species and habitat quality of the nest. If new off-bouts were manually added, they followed the program criteria: minimum temperature drop of 1 °C and minimum off-bout length of 4 min. To get accurate estimates of incubation temperature and length of the trips to nest, and because the program does not automatically mark on-bouts, these selections were manually added (seen as constant or increasing temperature between off-bouts). The nocturnal incubation session was determined to be the period between the start of the last on-bout in the evening and the first off-bout of the following morning.

Because loggers were usually placed into the nests around midday, we ensured the equal length of the temperature data sets by discarding the first recording day and by selecting the following 48-h period for analyses (i.e. days 2 and 3 when the logger was in the nest). This 48-h period started from the first off-bout of the second day and ended after the nocturnal incubation session of the third day. Consequently, the selected 48-h period included the incubation days 1–2 (63%, 28 out of 44 nests), or days 3–4 (27%, 11 out of 44 nests, the rest being 0, 3rd or 5th incubation day, this included 4 nests out of 44) in the early incubation session. For the middle incubation session, the 48-h period included incubation days 6–7 (60%, 25 out of 44) or days 7–8 (22.7%, the rest being days 5 or 8 of incubation). From these 48-h incubation data, we followed the methods of Nord and Nilsson (2012) and calculated the total number of off-bouts, average off-bout and on-bout durations and temperatures, and the average length of the nocturnal incubation session for both incubation periods. Furthermore, incubation constancy, i.e. the percentage of the daytime spent incubating, was calculated by dividing the time spent actively incubating by the total time spent both incubating and away from the nest (excluding the nocturnal incubation session). In addition, we calculated the temperature decrease during each off-bout, i.e. off-bout heat loss, by subtracting the minimum temperature from the maximum temperature (i.e. temperature before the female left the nest minus the lowest temperature during the off-bout). We also calculated the average temperature during the 48-h observation period (including off- and on-bouts as well as nocturnal incubation sessions) separately for the early and middle incubation periods (Nord and Nilsson 2012) to describe the thermal environment experienced by the embryos, hereafter referred to as “nest temperature”.

As several studies have demonstrated the importance of ambient temperature on avian incubation behaviour (e.g. Conway and Martin 2000; McClintock et al. 2014; Walters et al. 2016), we also used measurements from the nearest weather station (Kalmar) of the Swedish Meteorological and Hydrological Institute, from 2014 (downloaded at https://opendata-download-metobs.smhi.se/explore/#) as a covariate in our models (see below). Based on the hourly temperature measurements, we calculated the average temperature between 4 am to 9 pm (i.e. daytime activity of the birds based on our incubation data) for the 48-h incubation period for each nest separately for the early and middle incubation sessions. Thus, the ambient temperature measurements we used reflected the outside temperature during the time of day when birds were actively moving in and out of the nest.

### Statistical analyses

For all analyses, we used SAS (v 9.4.) statistical software and the Kenward-Roger method (latest version, Kenward and Roger 2009) to compute the degrees of freedom. In the incubation analyses, 41 out of the 44 nests (28 collared and 13 pied flycatcher nests, out of which four pied flycatcher nests located in pine forests) were used, as three nests were missing habitat quality estimates. In the hatching analyses, 36 nests (25 collared and 11 pied flycatcher nests) were used due to missing hatching data values.

#### Incubation data

We analysed the number of off-bouts, average off-bout and on-bout durations, average on-bout temperature, incubation constancy (percentage of daytime spent incubating), average length of the nocturnal incubation sessions, average off-bout heat loss (temperature drop during off-bout) and nest temperature (average temperature during the 48 h across on- and off-bouts) of each nest as dependent variables in similar generalized linear mixed models (GLMM). In these GLMMs, we included female species, incubation stage (early or middle), habitat quality (frass mg/day/m^3^), ambient temperature, clutch size and the pairwise interactions between species and habitat quality as explanatory variables. Due to the variation when the logger was placed in to the nest, the incubation day from which the recording of the 48-h incubation behaviour started (which was highly correlated with the lay date, see the “Methods” section) was initially added as a covariate in all models, but removed from the final models as it did not significantly affect our variables of interest. Similarly, the consistently non-significant interactions between species and habitat quality were left out from the final models to avoid overparametrization, starting the step-wise selection from the interaction. The number of off-bouts was analysed with a Poisson error distribution and a log link function, while off-bout duration, nest temperature, off-bout heat loss and the length of nocturnal incubation sessions were analysed with a lognormal error distribution and an identity link function. On-bout temperature was analysed with a normal distribution (normality checked from residuals) and incubation constancy was analysed with a beta error distribution and a logit link function. Nest was set as a random effect to control for the correlation structure of the dataset in all models.

#### The effect of incubation behaviour on the length of incubation and hatching probability

Due to the limited data set and low variation in the length of incubation (hatching date − (lay date+ clutch size)), we used non-parametric Spearman correlations between the length of incubation and number of off-bouts, on- and off-bout duration, ambient temperature, on-bout and nest temperatures and habitat quality. This was done separately for each incubation stage. The probability of hatching was analysed with a GLMM with a binomial error distribution and a logit link function with event/trials syntax, where events were the number of hatched nestlings and trials were clutch size. Average nest temperature across the two incubation periods (representing overall thermal environment in the nest across the two during incubation) as well as the species of female were set as explanatory variables.

## Results

### Incubation data

Out of the 13 pied flycatcher females, 71.4% were nesting in the same forest with collared flycatchers, whereas the other 28.6% where in areas without collared flycatchers. In this sample of breeding pairs, the average habitat quality was similar in both collared and pied flycatcher territories across the two incubation stages (raw data presented in Supplement 1, Fig. S1). The average ambient daytime temperature across the 48-h measuring period was 15.2 ± SD 3.19 °C and 13.1 ± SD 2.33 °C for the early and middle incubation periods, respectively. For all GLMM models, model estimates, standard errors, degrees of freedom, and *t* values for all explanatory and random variables are presented in Supplement 1 (Tables S1–S9, back-transformed values for estimates of the covariates are presented in Table S10).

**Fig. 1:**
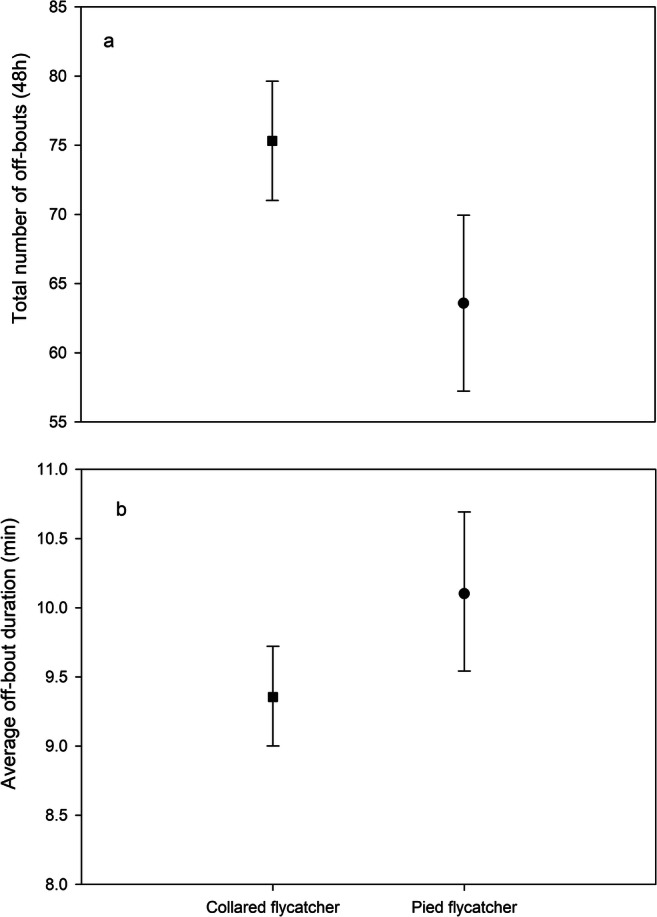
Result from GLMM (mean and 95% CIs) explaining differences in **a** the number of off-bouts and **b** average off-bout duration between collared flycatchers (square) and pied flycatchers (circle) over the 48-h observation period

**Fig. 2:**
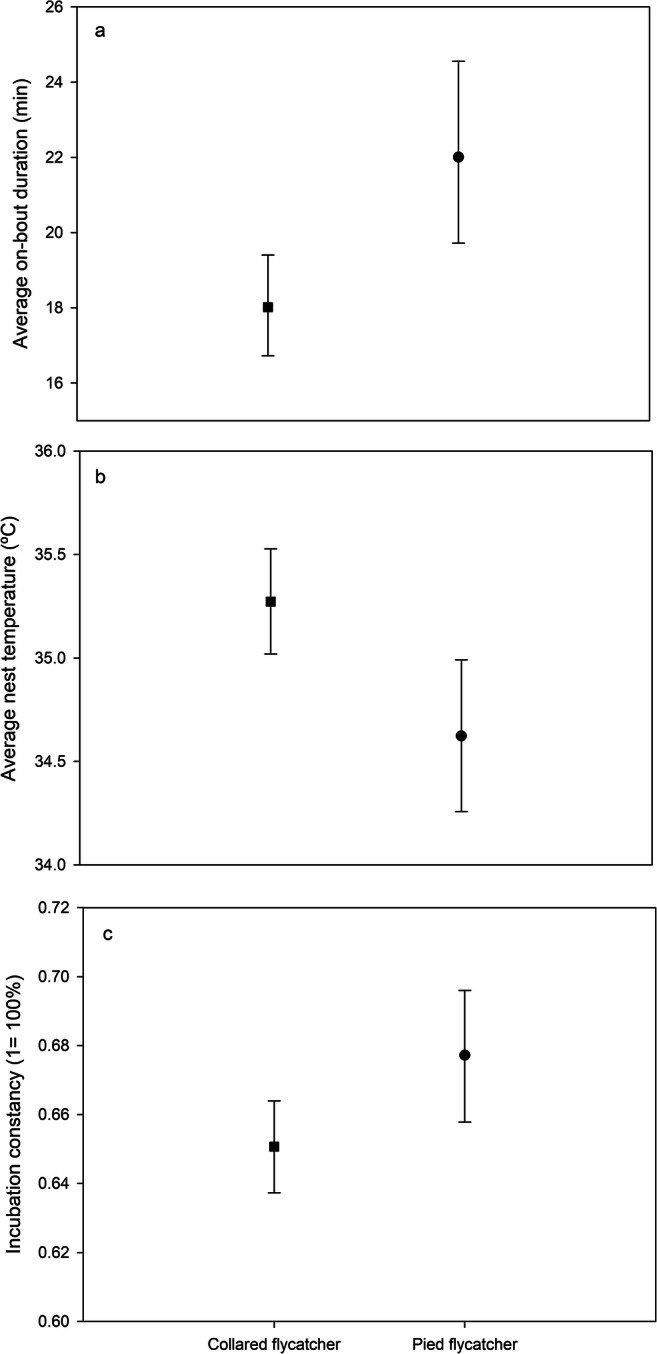
Result from GLMM (mean and 95% CIs) explaining the differences in **a** average on-bout duration, **b** nest temperature and **c** incubation constancy (percentage of daytime spent incubating) between collared flycatchers (square) and pied flycatchers (circle) over 48 h observation period

Pied flycatchers had significantly fewer and longer off-bouts compared to collared flycatchers (Table 1, Fig. 1a, b), resulting in longer on-bouts and higher incubation constancy (Table 1, Fig. 2a). There was no significant difference (Table 1) between species in off-bout heat loss (collared flycatcher: mean 2.709 ± SE 1.038 °C; pied flycatcher: mean 2.909 ± SE 1.056 °C). Despite this, collared flycatchers had a significantly higher on-bout temperature (mean 35.520 °C, 95% CIs 35.285 to 35.756 °C) compared to pied flycatchers (mean 34.892 °C, 95% CIs 34.544 to 35.240 °C, Table 1), which also resulted in a higher nest temperature in collared flycatcher nests (Table 1, Fig. 2b).

**Table 1:** Effect of species (collared and pied flycatcher), ambient temperature, stage of incubation (early or middle), habitat quality (caterpillar frass mg/day/m^3^) and clutch size on the number of off-bouts (foraging trips out of the nest), average off- and on-bout bout durations, off-bout heat loss (average change in temperature during off-bouts), average on-bout and nest temperature (temperature during incubation and nest temperature during the 48-h period including off-and on-bouts, respectively), incubation constancy (percentage of the daytime spent incubating) and the average length of nocturnal incubation session

**Independent variables**	Number of off-bouts		Off-bout duration		Off-bout heat loss		On-bout duration		On-bout temperature		Nest temperature		Incubation constancy		Nocturnal incubation session	
	*F*_df_	*P*	*F*_df_	*P*	*F*_df_	*P*	*F*_df_	*P*	*F*_df_	*P*	*F*_df_	*P*	*F*_df_	*P*	*F*_df_	*P*
Species	10.3 1, 38.87	0.003	5.09 1,33.98	0.031	1.14 1,38.23	0.293	9.24 1,37.55	0.004	9.07 1,36.85	0.005	8.61 1,36.32	0.006	5.11 1,37.95	0.030	1.02 1,38.03	0.320
Temperature	7.07 1,76.00	0.010	9.08 1,51.04	0.004	11.65 1,50.33	0.001	6.58 1,50.01	0.013	41.06 1,52.34	< 0.001	37.71 1,50.48	< 0.001	0.38 1,53.7	0.543	1.81 1,57.19	0.184
Incubation stage	11.88 1,76.00	0.001	40.33 1, 35.7	< 0.001	3.76 1,39.37	0.060	6.41 1,38.73	0.016	40.36 1,38.42	<0.001	34.68 1,37.70	<0.001	3.04 1,39.25	0.089	0.93 1,40.17	0.340
Habitat quality	6.77 1,76.00	0.011	0.87 1,74.73	0.353	0.80 1,68.49	0.373	6.55 1,68.86	0.013	9,81 1,73.66	0.003	7.77 1,71.51	0.007	3.56 1,74.57	0.063	0.11 1, 76.00	0.739
Clutch size	10.15 1,36.74	0.003	12.70 1,35.11	0.001	12.59 1,38.93	0.001	6.35 1,38.28	0.016	6.08 1,37.87	0.018	7.09 1,37.19	0.011	0.54 1,38.7	0.469	1.52 1,39.49	0.225

The number of off-bouts decreased, whereas the average on-bout duration increased with increasing habitat quality (Tables 1, S1 and S4). Furthermore, both on-bout and nest temperatures increased with increasing habitat quality values (Tables 1, S5 and S7). Habitat quality had no significant effect on the average off-bout duration or off-bout heat loss (Tables 1, S2 and S3). However, there was a positive trend (Table 1), suggesting that the incubation constancy tended to increase with increasing habitat quality (Table S6).

During the early stage of incubation, birds took significantly fewer (mean 65.331, 95% CIs 61.538 to 69.359 trips) but longer off-bouts (mean 10.475 min, 95% CIs 10.054 to 10.913 min) compared to the middle stage of incubation (number of off-bouts mean 72.025, 95% CIs 67.928 to 76.369 trips; average off-bout duration mean 9.021 min, 95% CIs 8.659 to 9.397 min) during the observed 48-h period (Table 1). This also resulted in significantly longer on-bout durations (Table 1) during the earlier stage of incubation (mean 20.866 min, 95% CIs 19.362 to 22.484 min) compared to the middle incubation stage (mean 19.001 min, 95% CIs 17.635 to 20.475 min). There was no significant difference in off-bout heat loss or incubation constancy between the two incubation periods (Table 1). However, both on-bout and hence nest temperatures were significantly higher (Table 1) during the middle stage of incubation (on-bout temperature: mean 35.639, 95% CIs 35.393 to 35.886; nest temperature: mean 35.347 °C, 95% CIs 35.086 to 35.605 °C) compared to the earlier incubation stage (on-bout temperature: 34.773 °C, 95% CI 34.526 to 35.019 °C; nest temperature mean 34.550 °C, 95% CIs 34.295 to 34.803 °C).

The number of off-bouts decreased with increasing ambient temperature and clutch size, whereas off-bout duration increased with ambient temperature and clutch size (Tables 1, S1 and S2). Off-bout temperature (i.e. heat loss from the eggs) decreased with increasing ambient temperature and clutch size (Tables 1 and S3). Neither ambient temperature nor clutch size affected the incubation constancy (Tables 1 and S6).

However, on-bout duration as well as on-bout and nest temperatures increased with ambient temperature (Tables 1, S4, S5 and S7). On-bout duration also significantly increased with clutch size, whereas on-bout and nest temperature decreased with clutch size (Tables 1, S4, S5 and S7).

None of the explanatory factors affected the length of nocturnal incubation session (Tables 2 and S8).

**Table 2:** Results from a GLMM for species (collared or pied flycatcher) and average nest temperature across the two incubation stages (early and middle) explaining variation in probability of hatching (*N* = 36 nests)

	Probability of hatching	
	*F*_df_	*P*
Species	0.84 _1,33_	0.37
Average nest temperature across incubation stages	0.38 _1,33_	0.54

### The effect of incubation behaviour on the length of incubation and hatching probability

During the early stage of incubation, the length of incubation was negatively correlated with on-bout temperature (Spearman correlation coefficient − 0.34, *P* = 0.03), and during the later stage of incubation the trend was similar (− 0.28, *P* = 0.07). There was also a trend towards a negative correlation between the length of incubation and nest temperature (Spearman correlation coefficient − 0.29, *P* = 0.06 for both incubation stages), whereas the number of off-bouts, on- or off-bout length, ambient temperature or habitat quality were not correlated with length of incubation (all *P* > 0.1). The species did not differ in incubation length (ANOVA, *F*_1, 40_ = 2.99, *P* = 0.09).

In 89% of the nests, all the eggs hatched. There was difference neither between species (collared flycatcher: mean 0.98%, 95% CIs 0.94 to 0.99%; pied flycatcher: mean 0.95%, 95% CIs 0.86 to 0.99%) nor an effect of nest temperature on hatching probability (Tables 2 and S9).

## Discussion

We found that both pied and collared flycatcher females endured higher nest attendance and incubation constancy when they were breeding in territories with higher caterpillar availability. There were, however, some key differences in the incubation behaviour of these two sympatric flycatcher species. The competitively more dominant collared flycatcher, whose nestlings are more sensitive to food shortages, made more frequent foraging trips but also allocated more heat to their eggs, leading to lower nest attendance and yet higher nest temperature compared to pied flycatchers. Below, we discuss our findings in the light of niche separation and highlight the importance of female reproductive behaviours in setting the stage for differences in niche requirements of species competing for similar resources.

The increasing caterpillar availability during incubation seems to have relaxed the trade-off between self-maintenance and embryonic needs for both species. Prey capture rates of both flycatcher species should be higher in territories with high food availability (Adamíc and Bureš 2007), which likely explains the observed lower number of off-bouts seen among females breeding in such territories. A lower number of off-bouts, in turn, results in increased nest attendance, higher incubation constancy and thereby overall higher and more constant nest temperature, which is favourable for the developing embryos. Food supplementation studies further support the importance of territory quality for explaining variation in incubation behaviour: access to supplementary food increases the nest attendance of Australian reed warbler (*Acrocephalus australis*) (Eikenaar et al. 2003), and reduces the length of off-bouts and increases incubation constancy in karoo prinia (*Prinia maculosa*) (Chalfoun and Martin 2007). The effect seems to be similar in biparentally incubating species, such as the silvereye (*Zosterops lateralis*) (Barnett and Briskie 2010). Although males can support incubating females by feeding them on the nest during harsh conditions such as during periods of poor food availability (Lifjeld and Slagsvold 1986; Cantarero et al. 2014; Amininasab et al. 2016; Kötél et al. 2016) also in species where only the females incubates the eggs. However, as increase in food availability was associated with increased nest attendance, the aid provided by the males was likely minor, or did not fully compensate for differences in food availability between habitats in our study.

We found that both species were successfully able to balance between self-maintenance and embryonic needs across habitat types, maintaining the temperature of the eggs well above the physiological zero temperature of 25–27 °C (Haftorn 1988). This resulted in high hatching success across nests with complete hatching success in 89% of the nests. However, collared flycatcher females needed more frequent off-bouts across habitat types indicating that they may indeed be more sensitive to food limitation. This resulted in lower incubation constancy in collared flycatcher nests compared to pied flycatchers. Despite this, collared flycatchers were able to reach higher nest temperature indicating that they compensated the lower nest attendance by higher heat exchange during incubation. More frequent off-bouts together with higher on-bout temperatures suggest that the difference in incubation behaviour maybe be a result of (1) higher energetic needs of collared flycatcher females, (2) differences in hunting efficiency and/or (3) prey preference between the two species.

There is some support for interspecific differences in energetic needs: collared flycatcher nestlings are less able to adjust their resting metabolic rate to match current environmental conditions compared to pied flycatcher nestlings (McFarlane et al. 2018), which is likely a key reason why the breeding of collared flycatchers is limited to high-quality territories (Qvarnstörm et al. 2005, 2007; Rybinski et al. 2016; McFarlane et al. 2018; Sirkiä et al. 2018). Conversely, no differences in resting metabolic rate have been observed between adult males of the two species (McFarlane et al. 2016). However, potential interspecific differences in metabolic rates of incubating females have not been tested.

More frequent but shorter off-bouts may also indicate that collared flycatchers are less effective at hunting, perhaps collecting fewer prey items per trip, thus requiring more frequent foraging trips. Another option is that collared flycatchers have a relatively stronger preference for specific prey (such as caterpillars) compared to pied flycatchers. Although the diets of the two species are overlapping (Bureš 1995; Wiley et al. 2007), there is some interspecific variation in foraging preferences. For example, pied flycatchers are more likely to catch prey requiring higher flight manoeuvrability, such as flying insects (Bureš 1995). Although both species also hunt prey from leaves and trunks, pied flycatchers are more likely to forage also on the ground, whereas collared flycatchers prefer to forage in the canopy (Bureš 1995; Adamíc and Bureš 2007). These three possible explanations (differences in female energetic needs, preference for specific prey or ability to catch non-caterpillar prey) for observed differences in nest attendance behaviour are not mutually exclusive. Further, possible differences in energetic needs between the species may result in divergent foraging behaviour.

A final possibility, which could interact with the three proposed mechanisms above, is that the higher temperature sensitivity of developing collared flycatcher embryos forces the females to keep the nest temperature high by releasing more heat. We found some support for this, as despite the differences in nest attendance, collared flycatchers reached higher incubation temperatures, but this difference did not result in higher hatching success. Therefore, collared flycatcher embryos may indeed require higher temperature for successful development. Although long-term effects of incubation temperature are not well known, there is some evidence showing that higher nest temperature and incubation constancy may affect offspring survival, metabolism, size or growth later in life (Kim and Monaghan 2006; Hulet et al. 2007; Nord and Nilsson 2011; Hepp and Kennamer 2012; Wada et al. 2015; Berntsen and Bech 2016, but see Nord and Nilsson 2016). Therefore, differences in female incubation behaviours, nest temperature and embryonic needs be tightly evolutionary intertwined with interspecific differences in competitive abilities and niche use of the two flycatcher species. Manipulative experiments are nevertheless needed to fully disentangle whether the two species differ in energetic needs, prey catching abilities or prey preference during incubation and/or thermal sensitivity of embryos and to what extend these differences contribute to the outcomes of interspecific competition.

Our results also tentatively suggest that the higher incubation temperature found during the early stage of incubation may shorten the overall time needed for incubation, indicating that this stage of incubation may be an especially important period for embryo development. A similar pattern has been documented in herring gulls (*Larus argentatus*), where higher incubation constancy during the early stage of incubation sped up the development and improved the quality of the offspring (Kim and Monaghan 2006). Therefore, food availability in the breeding habitat is likely one of the key factors influencing offspring development and/or quality by affecting the trade-off between self-maintenance and embryo care.

To conclude, our results indicate that even sympatric and closely related species, such as collared and pied flycatchers, can show differences in incubation behaviour, leading to differences in nest temperature. As the thermal environment of the nest can affect several nestling characteristics, incubation behaviour may be one of the factors influencing the ability of offspring to withstand environmental changes. Therefore, differences in incubation behaviour may partly mitigate interspecific competition of sympatric, ecologically similar species. Future studies are required to assess the physiological differences between collared- and pied flycatchers, and to test to what extent the observed differences in incubation behaviour contribute to interspecific difference in tolerance to food shortages. Our results also support previous findings demonstrating that high food availability during incubation can relax the trade-off between incubation and self-maintenance, and increase nest attendance and nest temperature, highlighting the importance of territory quality for early parental investment.

## Electronic supplementary material

ESM 1 (DOCX 55.7 kb)

## Data Availability

The datasets generated and/or analysed during the current study are available from the corresponding author on reasonable request.
