# Supplementary material for: Differences in incubation behaviour and niche separation of two competing flycatcher species
Source: Behav Ecol Sociobiol. 2020 Aug 1;74(8):105. doi: 10.1007/s00265-020-02883-4 (PMC7410113; doi:10.1007/s00265-020-02883-4)
Supplement: Supplementary file 1 — (DOCX 55.7 kb) [file 265_2020_2883_MOESM1_ESM.docx]

Behavioral Ecology and Sociobiology

Differences in incubation behaviour and niche separation of two competing flycatcher species

Tuuli-Marjaana Koski*^1,2,^, Päivi M. Sirkiä^1,3^, S. Eryn McFarlane^4,5^, Murielle Ålund^6^, Anna Qvarnström^7^

*Corresponding author:

Tuuli-Marjaana Koski; tmhkos@utu.fi

Tel +358 40 7719168

^1^Department of Biology and Biodiversity unit, University of Turku, FI-20014, Turku, Finland

^2^Integrated Plant Protection Unit, Department of Plant Protection Biology, Swedish University of Agricultural Sciences, 23053 Alnarp, Sweden.

^3^Finnish Museum of Natural History, Zoology Unit, P.O. Box 17, FI-00014, University of Helsinki, Finland

^4^Institute of Evolutionary Biology, University of Edinburgh, Charlotte Auerbach Road, Edinburgh EH9 3FL, United Kingdom

^5^Biological Sciences, Lund University, Sölvegatan 37, 223 62 Lund, Sweden

^6^Department of Integrative Biology, Michigan State University, 288 Farm Lane, East-Lansing, 48824, USA

^7^Animal Ecology, Department of Ecology and Genetics, Uppsala University, Norbyvägen 18d, 752 36 Uppsala, Sweden

Supplement 1

Figure S1 presenting habitat quality (caterpillar frass mg/d/m^3^) overlaps across of collared (blue) and pied flycatcher (orange) territories (raw data) during both early and late incubation stages. Each dot represent the average habitat quality for individual nest.


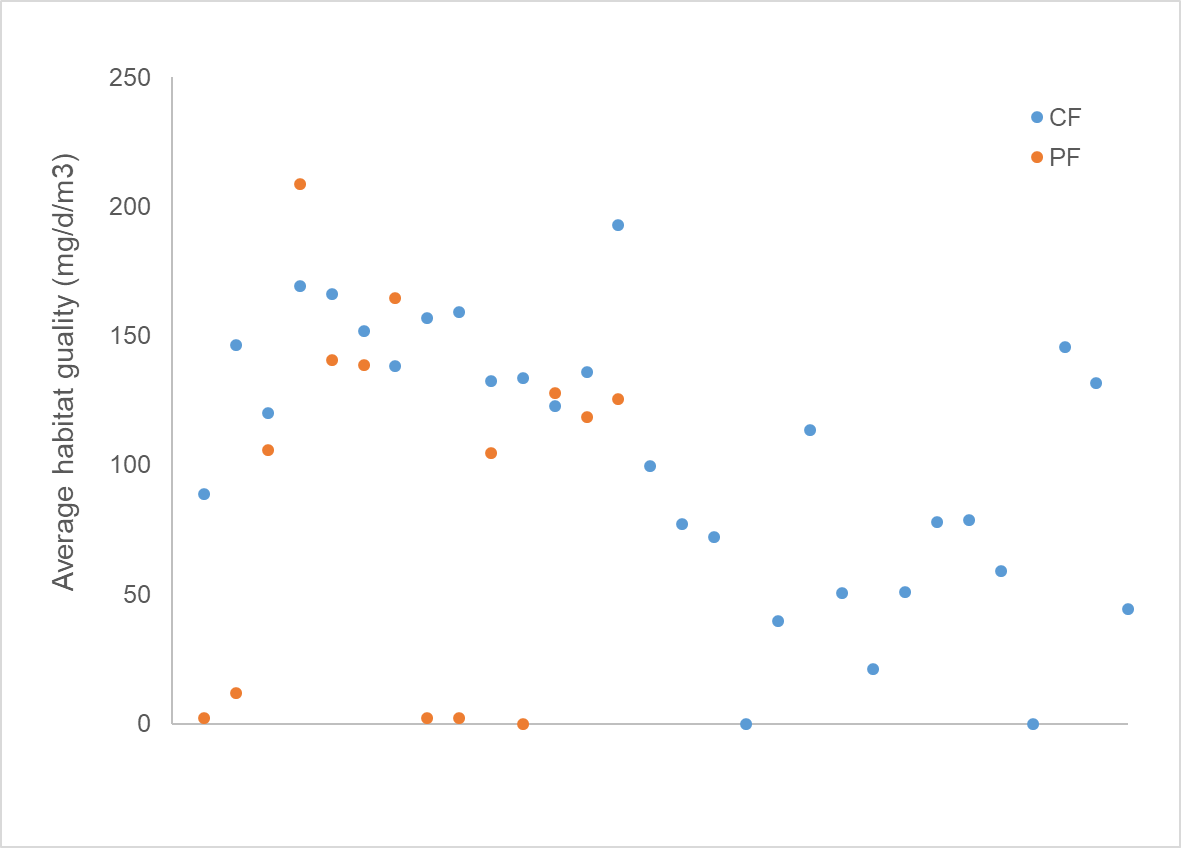


Model estimates (Tables S1- S9, not back-transformed), standard errors, degrees of freedoms and t-values for the explanatory and random variables (nest) for models of incubation behavior and hatching success. If not otherwise mentioned, the estimates are given on a log scale.

**Table S1** Result from GLMM for variables explaining the **total number of off-bouts** (visits out of the nest) on collared and pied flycatcher.

| *Independent variables* | Estimate | SE | df | t value |
| --- | --- | --- | --- | --- |
| Intercept | 5.2566 | 0.2529 | 41.85 | 20.79 |
| Sp. female (collared flycatcher) | 0.1669 | 0.0527 | 38.87 | 3.17 |
| Ambient temperature | -0.01532 | 0.005764 | 76 | -2.66 |
| Incubation stage (early) | -0.09754 | 0.02831 | 76 | -3.45 |
| Habitat quality | -0.00068 | 0.000262 | 76 | -2.6 |
| Clutch size | -0.1169 | 0.03670 | 36.74 | -3.19 |
| *Random effect* | | | | |
| Nest | 0.01642 | 0.005434 |  | |

**Table S2** Result from GLMM model for factors explaining **off-bout duration** (length of foraging trips) on collared and pied flycatchers

| *Independent variables* | Estimate | SE | df | t value |
| --- | --- | --- | --- | --- |
| Intercept | 1.4452 | 0.17 | 40.79 | 8.5 |
| Sp. female (collared flycatcher) | -0.07684 | 0.03407 | 33.98 | -2.26 |
| Ambient temperature | 0.01416 | 0.004698 | 51.04 | 3.01 |
| Incubation stage (early) | 0.1495 | 0.02354 | 35.7 | 6.35 |
| Habitat quality | 0.000188 | 0.000201 | 74.73 | 0.93 |
| Clutch size | 0.08641 | 0.02425 | 35.11 | 3.56 |
| *Random effect* | | | | |
| Nest | 0.005184 | 0.002793 |  | |

**Table S3** Result from GLMM model for variables explaining **off-bout heat loss** (temperature drop during off-bout) on collared and pied flycatchers

| *Independent variables* | Estimate | SE | df | t value |
| --- | --- | --- | --- | --- |
| Intercept | 2.5499 | 0.3267 | 43.98 | 7.8 |
| Sp. female (collared flycatcher) | -0.07113 | 0.06673 | 38.23 | -1.07 |
| Ambient temperature | -0.02582 | 0.007565 | 50.33 | -3.41 |
| Incubation stage (early) | -0.07143 | 0.03685 | 39.37 | -1.94 |
| Habitat quality | 0.000304 | 0.000339 | 68.49 | 0.9 |
| Clutch size | -0.168 | 0.04736 | 38.93 | -3.55 |
| *Random effect* | | | | |
| Nest | 0.02665 | 0.009177 |  | |

**Table S4** Result from GLMM model for variables explaining **on-bout duration** (length of incubation sessions between off-bouts) on collared and pied flycatchers

| *Independent variables* | Estimate | SE | df | t value |
| --- | --- | --- | --- | --- |
| Intercept | 1.8986 | 0.3225 | 43.4 | 5.89 |
| Sp. female (collared flycatcher) | -0.2 | 0.06579 | 37.55 | -3.04 |
| Ambient temperature | 0.01942 | 0.007569 | 50.01 | 2.57 |
| Incubation stage (early) | 0.09352 | 0.03693 | 38.73 | 2.53 |
| Habitat quality | 0.000867 | 0.000339 | 68.86 | 2.56 |
| Clutch size | 0.1177 | 0.0467 | 38.28 | 2.52 |
| *Random effect* | | | | |
| Nest | 0.02552 | 0.009071 |  | |

**Table S5** Result from GLMM model for variables explaining **on-bout temperature** (temperature during incubation) on collared and pied flycatchers (estimate on a normal scale).

| *Independent variables* | Estimate | SE | df | t value |
| --- | --- | --- | --- | --- |
| Intercept | 34.8498 | 1.0361 | 43.42 | 33.64 |
| Sp. female (collared flycatcher) | 0.6287 | 0.2087 | 36.85 | 3.01 |
| Ambient temperature | 0.1756 | 0.0274 | 52.34 | 6.41 |
| Incubation stage (early) | -0.8666 | 0.1364 | 38.42 | -6.35 |
| Habitat quality | 0.0037 | 0.0012 | 73.66 | 3.13 |
| Clutch size | -0.3659 | 0.1484 | 37.87 | -2.47 |
| *Random effect* | | | | |
| Nest | 0.2139 | 0.0961 |  | |

**Table S6** Result from GLMM model for variables explaining **incubation constancy** (percentage of daytime spent incubating) (results on logit scale)

| *Independent variables* | Estimate | SE | df | t value |
| --- | --- | --- | --- | --- |
| Intercept | 0.4695 | 0.2604 | 44.37 | 1.8 |
| Sp. female (collared flycatcher) | -0.1184 | 0.05237 | 37.95 | -2.26 |
| Ambient temperature | 0.004321 | 0.007055 | 53.7 | 0.61 |
| Incubation stage (early) | -0.06177 | 0.03545 | 39.25 | -1.74 |
| Habitat quality | 0.000574 | 0.000304 | 74.57 | 1.89 |
| Clutch size | 0.02722 | 0.03718 | 38.7 | 0.73 |
| *Random effect* | | | | |
| Nest | 0.0126 | 0.00599 |  | |

**Table S7** Result from GLMM model for **nest temperature** (average temperature across off- and on-bouts during the 48h observation period)

| *Independent variables* | Estimate | SE | df | t value |
| --- | --- | --- | --- | --- |
| Intercept | 3.5563 | 0.03131 | 42.59 | 113.58 |
| Sp. female (collared flycatcher) | 0.01861 | 0.006343 | 36.32 | 2.93 |
| Ambient temperature | 0.004824 | 0.000786 | 50.48 | 6.14 |
| Incubation stage (early) | -0.0228 | 0.003872 | 37.7 | -5.89 |
| Habitat quality | 0.000096 | 0.000035 | 71.51 | 2.79 |
| clutch size | -0.012 | 0.004507 | 37.19 | -2.66 |
| *Random effect* | | | | |
| Nest | 0.000217 | 0.000088 |  | |

**Table S8** Result from GLMM model for the **length of nocturnal incubation session**

| *Independent variables* | Estimate | SE | df | t value |
| --- | --- | --- | --- | --- |
| Intercept | 6.0497 | 0.1436 | 45.21 | 42.13 |
| Sp. female (collared flycatcher) | -0.02871 | 0.02848 | 38.03 | -1.01 |
| Ambient temperature | -0.00583 | 0.004335 | 57.19 | -1.35 |
| Incubation stage (early) | -0.02149 | 0.02225 | 40.17 | -0.97 |
| Habitat quality | -0.00006 | 0.000178 | 76 | -0.33 |
| clutch size | 0.02507 | 0.02031 | 39.49 | 1.23 |
| *Random effect* | | | | |
| Nest | 0.002669 | 0.001869 |  | |

**Table S9** Result from GLMM for species (Sp. female) and nest temperature (temperature across off- and on-bouts during 48h) as an average for early and middle incubation period explaining variation in hatching success (estimates on logit scale)

| *Independent variables* | Estimate | SE | df | t value |
| --- | --- | --- | --- | --- |
| Intercept | 14.7826 | 18.9386 | 33 | 0.78 |
| Sp. female (collared flycatcher) | 0.7445 | 0.812 | 33 | 0.92 |
| Average nest temperature | -0.3345 | 0.5416 | 33 | -0.62 |

**Table S10** Back transformed estimates as percentages ± SE from GLMM explaining the effect of habitat quality (caterpillar frass mg / d/ m^3^), ambient temperature, and clutch size on the number of off-bouts (foraging trips out of the nest), off- and on-bout bout durations, off-bout heat loss (change in temperature during off-bout), on-bout and nest temperature (temperature during incubation and nest temperature during the 48 h period including off-and on-bouts, respectively), incubation constancy (percentage of the daytime spent incubating) and the length of nocturnal incubation session. Asterisk (*) indicates statistical significant difference reported in the manuscript Table 1. **Note** that estimates for on-bout temperature did not require back-transformation and is presented on a normal scale (marked as ^norm^), whereas estimates for incubation constancy are back transformed from logit scale and should thus be interpret as proportional odds ratio (marked as ^odds^).

|  | Number of off-bouts  (± SE) | Off-bout duration  (± SE) | Off-bout heat loss  (± SE) | On-bout duration  (± SE) | On-bout temperature^norm^  (± SE) | Nest temperature  (± SE) | Incubation constancy^odds^  (± SE) | Nocturnal incubation session( ± SE) |
| --- | --- | --- | --- | --- | --- | --- | --- | --- |
| Habitat quality | -0.068*± 0.026 | 0.019 ± 0.020 | 0.030 ± 0.034 | 0.087*± 0.034 | 0.004* ±  0.001 | 0.010* ± 0.004 | 0.500 ± 0.500 | -0.006 ± 0.018 |
| Ambient temperature | -1.520* ± 0.578 | 1.426* ± 0.471 | -2.549* ± 0.759 | 1.961*± 0.760 | 0.176* ± 0.027 | 0.484*± 0.079 | 0.501 ± 0.502 | -0.581 ± 0.434 |
| Clutch size | -11.033* ± 3.738 | 9.025* ± 2.455 | -15.465*± 4.850 | 12.491* ± 4.781 | -0.366* ± 0.148 | -1.193* ± 0.452 | 0.507 ± 0.509 | 2.539 ±  2.052 |
